# Helicity dependent photoresistance measurement vs. beam-shift thermal gradient

**DOI:** 10.1038/s41467-022-34198-5

**Published:** 2022-11-10

**Authors:** Haozhe Yang, Eva Schmoranzerová, Pyunghwa Jang, Jayshankar Nath, Thomas Guillet, Isabelle Joumard, Stéphane Auffret, Matthieu Jamet, Petr Němec, Gilles Gaudin, Ioan-Mihai Miron

**Affiliations:** 1 Univ. Grenoble Alpes, CNRS, CEA, SPINTEC, F-38000 Grenoble, France; 2 Faculty of Mathematics and Physics, Charles University, Prague, Czech Republic; 3 Present Address: CIC nanoGUNE BRTA, 20018 Donostia-San Sebastian, Basque Country Spain

**Keywords:** Magneto-optics, Spintronics

## Abstract

Optical detection techniques are among the most powerful methods used to characterize spintronic phenomena. The spin orientation can affect the light polarization, which, by the reciprocal mechanism, can modify the spin density. Numerous recent experiments, report local changes in the spin density induced by a circularly polarized focused laser beam. These effects are typically probed electrically, by detecting the variations of the photoresistance or photocurrent associated to the reversal of the light helicity. Here we show that in general, when the light helicity is modified, the beam profile is slightly altered, and the barycenter of the laser spot is displaced. Consequently, the temperature gradients produced by the laser heating will be modulated, producing thermo-electric signals that alternate in phase with the light polarization. These unintended signals, having no connection with the electron spin, appear under the same experimental conditions and can be easily misinterpreted. We show how this contribution can be experimentally assessed and removed from the measured data. We find that even when the beam profile is optimized, this effect is large, and completely overshadows the spin related signals in all the materials and experimental conditions that we have tested.

## Abstract (web summary)

Many recent studies have explored the response of magnetic systems to circularly polarised light. To achieve this, typically experiments use a birefringent crystal. Here, Yang et al show that any small error in the alignment of the crystal can result in a beam shift, and this shift can lead to spurious signals similar yet unrelated to the electron spin.

## Introduction

Spin based devices for information technology, such as magnetic memories and spin q-bits, rely on the ability to control and detect the spin orientation^1–3^. Optical manipulation and detection can be faster than electrical techniques while also easy to implement^4–6^. Unlike the electrical detection, it allows to extract pure spin-related information avoiding additional spurious effects caused by the proximity of the adjacent ferromagnet, or the interfacial spin scattering^7–11^. A non-vanishing spin density can cause helicity-dependent absorption of light or induce the rotation of the linear polarization^12–14^. This interaction, known as magnetic circular dichroism (or magneto-optical Kerr effect) has an inverse effect: the spin polarization is also perturbed by photons. It has been shown that circularly polarized^15^ light can induce the magnetization reversal^5,16^ as well as produce photovoltaic or photoresistive effects^17,18^. The changes of photoresistance^19–25^ or photocurrent^24,26–36^ have been attributed to the interaction between light and spin accumulation. This type of effect appears to be “universal”, as it has been observed in semiconductors^24,27^, semimetals^25,37^, topological insulators^29–36^, normal metals^19,20^ and even complex metal-organics structure^38^. However, its magnitude can differ by orders of magnitude even for similar materials and experimental conditions^30–32^. This significant discrepancy in the published results is indicative of at least one uncontrolled experimental parameter, related either to the material structure or to the measurement setup. Here we identify such a hidden parameter as a small beam shift that is generally produced when the light polarization is modified, interfering with the helicity-dependent spin signal.

The most common practical realization of experiments involving circularly polarized light, relies on the modulation of the light polarization by a birefringent crystal. The anisotropic index of refraction causes a different retardation of the orthogonal light polarizations, creating a relative phase shift that defines the resulting circular polarization. The left hand and right hand circularly polarized light is obtained by varying the angle between the optical axis of the birefringent crystal and the polarization plane of the linearly-polarized input light, either mechanically by rotating a quarter-wave plate (λ/4)^22,25,27–29,31,34–36,38^ or electrically by applying a high voltage to a photoelastic modulator^19–21,23,24,26,30,32,37^. The spin-related signals are extracted from the difference of the results obtained for the two helicities of the circularly polarized light.

The variation of the light helicity is obtained by modifying the polarization dependence of the refraction index somewhere in the optical path. From the perspective of the incoming linearly polarized light, it is as if the effective refraction index is varied. Any small geometrical imperfection or slight misalignment of the optical component that is used to perform this modification will cause a change of the illumination conditions. Therefore, in the case of a focused spot, the polarization change will always be associated to a small beam shift. The question is not whether the beam shift is present, but how large it is, and how important is its contribution to the measured signal, compared to the actual spin-dependent effect produced by the circularly polarized light.

To evaluate the interplay between these phenomena, we implemented similar detection schemes as in the original studies^19–21^, and focused on detecting the helicity-dependent photoresistance (HPR, this name will be used for spin-related photoresistance signal in this letter). We have tested several metallic layers and a topological insulator film in differently designed experimental setups under variable experimental conditions. In contrast to previous reports^19–21^, we do not observe any spin-dependent signal in any of our measurements. Instead, we identify the contribution from the unintended beam shift produced during light helicity modulation. This effect occurs under all experimental conditions: for different light intensities, different methods of polarization modulation, and different wavelengths. The beam shift modifies the local temperature gradients in the sample, creating thermo-electric signals that mimic the photovoltaic or photoresistive ones expected from the polarization modulation. Here, we characterize this additional effect, and we find a solution for correcting the results. Our findings are relevant for experiments where the circularly polarized focused beam is used to probe locally the helicity-dependent properties^19–24,26,29,31,34^, as the thermal gradients create similar signals. Furthermore, for experiments using a fixed circularly polarized beam^18,25,27,28,30,32,33,36–38^, the beam deflection associated to the helicity reversal may slightly modify the illumination conditions and also influence the results.

## Results and discussion

### Principle of HPR and beam shift-induced photoresistance

**Fig. 1:**
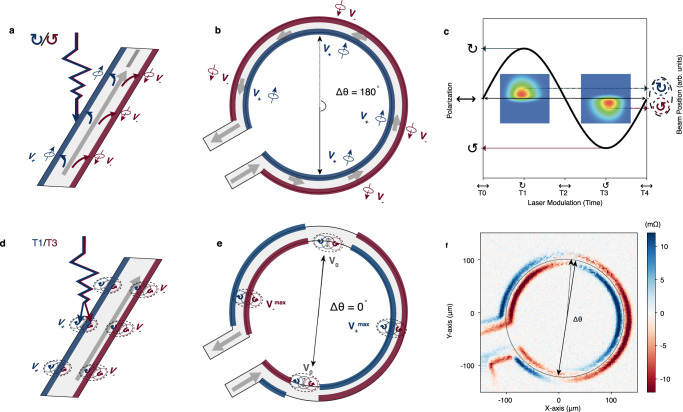
Spin accumulation induced photoresistance and beam shift induce photoresistance. Illustration of spin accumulation and the corresponding helicity dependent photoresistance detected for **a** a straight current path and **b** a ring shaped current path. The up/down spin accumulation appears at the edges of the device on the left/right side of the electric current. Thus, when illuminated by a circular polarized light, the absorption of one particular circularly-polarized light is different for spin-up and spin-down. The longitudinal resistance of the illuminated area on one edge will exhibit a higher value (blue) when illuminated with one particular helicity light, while on the other edge will be lower (red). **c** Cartoon illustration of the laser spot barycenter displacement associated to the polarization change from circular left (T1) to circular right (T3). The beam shift effect is illustrated by the two dashed circles the right y-axis, indicating the beam center positions are different at T1 and T3. An example of two laser spot profiles simulated with a 2D skewed Gaussian distribution are plotted in the insets. The laser spot with a different shape inherently will have a different effective position, which creates a different thermal gradient. To a first order, this effect can be approximated by a beam-shift. **d** The thermal gradients produced by the oscillating beam induce opposite longitudinal resistance variations at the two edges (blue and red). This heat gradient induced photoresistance is very similar to the helicity dependent photoresistance expected in the straight device. However, for the ring shaped device in **e**, these two effects have different symmetries: the contrast on the edges of the device changes sign along the circle. **f** Measured photoresistance mapping for 3 nm thick Pt ring shaped device. The angle difference Δ*θ* between the zero photoresistance position (dash arrow line) and the diameter across the other side of the zero photoresistance position (solid line) indicates the mixture of both spin accumulation effect *(Δθ* = 180°) and beam shift effect (Δ*θ* = 0°).

The principle of spin accumulation for the typical HPR measurement is illustrated in Fig. 1a. Let us assume that spin-up and spin-down are accumulated along each side of the current path due to spin orbital effect. The absorption of the left hand and right hand circularly-polarized light can be different for spin-up and spin-down, respectively. Thus, due to the slightly different absorption, the longitudinal resistance on one edge will be higher than the other edge when locally illuminated with the same circular light. The effect changes sign for opposite helicity.

The primary assumption for the HPR experiments is that the beam intensity and shape remain constant when the circular polarization is reversed. In this scenario, the photoresistance must result from the light-spin interaction. However, the laser beam is always distorted by some small amount when its polarization is modified. As this happens, the beam intensity may vary slightly, and the average beam position can be displaced, as shown in Fig. 1c. Particularly, the beam displacement plays a crucial role in generation of the parasitic signals. Due to a trivial laser heating, it induces a resistance variation at the same frequency as the circularly polarized light. The signal also changes signs between the two sample edges similarly to the HPR, as illustrated in Fig. 1d.

While these two effects appear to be indistinguishable in this particular geometry, their symmetry is generally not identical. Therefore, we can reliably separate them using a different sample geometry. Instead of a straight wire, we fabricate a ring-shaped device. Here, the effect of the HPR should be the same along the circular channel, as the spin-up and spin-down will be accumulated on the inner and outer side of the ring-shaped device (Fig. 1b). In contrast, the effect of the beam shift should exhibit a phase reversal as we move the laser along the circle (Fig. 1e) since the resulting signal is proportional to the projection of the beam shift on the sample edges. When the beam-shift is parallel to the edge, it produces no contribution to the signal. As the angle between the current and the beam shift axis is varied, the thermo-resistance reverses.

The experimental result of the 2D photoresistance mapping for the Pt ring device is shown in Fig. 1f. We observe two intersecting rings, following exactly the expectations from the beam shift-induced signal. If the experimental data included any contribution from the HPR, it should add to this effect, and the zero-crossings would no longer be at diametrically opposite positions on the ring, as shown in Fig. 1e. The relative contributions of the beam shift effect and HPR have been parametrized through an angle (Δθ in Fig. 1f), which quantifies the misalignment between the zero-crossings and the circle diameter. Δθ is 0° for pure beam shift-induced photoresistance, and becomes 180° for pure HPR. In our experiment, the measured Δθ is too small to be detected. A numerical simulation of the 2D photoresistance mapping with different HPR percentages is given in the Note 3 of the Supplementary Information. The simulation results show that Δθ increases with increasing the spin signal contributions to the total photoresistance. In our search for the HPR-related signal, we also measured systems such as Ta/SiO_2_, Cu/SiO_2_, and Pt/Yttrium Iron Garnet (YIG) (see Supplementary Information Note 5.1). The results are similar to those obtained for pure Pt, indicating that the beam shift-induced effect dominates the measured signal and no spin accumulation-induced HPR is detected.

### Transverse detection of the photoresistance for improved sensitivity

In order to further improve the sensitivity of the photoresistance detection, in a second experiment we use transverse resistance measurements in a Wheatstone bridge geometry. A symmetric Hall bar can behave similarly to a balanced Wheatstone bridge: the transverse voltage is always zero, despite the application of the electric current. When an off-centered laser spot is heating the Hall cross asymmetrically, the local change in resistivity due to the laser illumination will slightly deflect the current flow, which results in a transverse voltage. This behavior is very similar to an unbalanced Wheatstone bridge, where a change in one of the resistors gives rise to a transverse voltage signal as well.

**Fig. 2:**
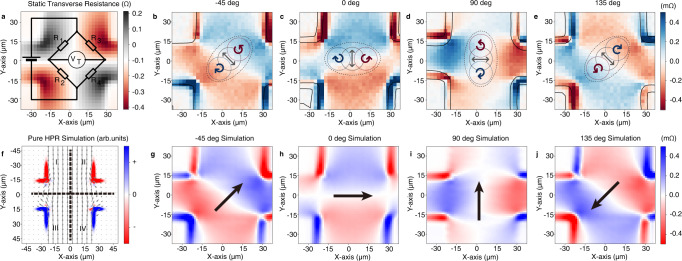
Beam shift-induced transverse photoresistance with a Wheatstone bridge model. **a** The static component of the 2D transverse resistance mapping of the Pt Hall cross can be viewed as a Wheatstone bridge with four resistors R1, R2, R3, and R4. The temperature rises in areas illuminated by the laser spot, which increases the resistance. The transverse voltage is positive when R1 or R4 increases, and negative value when R2 or R3 increases; **b**–**e** Transverse photoresistance mapping measured at different sample orientations. **f** An illustration of a simulated pure helicity-dependent photoresistance (HPR) effect, along with the charge current distribution shown as gray arrows. The spin accumulation is assumed to be proportional to the curl of the current along the transverse direction in a high-symmetrical material system with spin diffusion length much shorter than the laser spot. The transverse HPR effect is calculated by multiplying the distribution of spin accumulation with the Hall bar sensitivity function (i.e., the static transverse resistance mapping in **a**.) The Hall cross is split into four areas named I to IV. The current gradient is largest on the edge of the cross, where on the right edges of the cross (regions II and IV), HPR behaves similarly as in **a**, since the HPR acts in the same way as the laser heating. Vice versa, on the right edges (regions I and III) the HPR acts in the opposite way; **g**–**j** Photoresistance mapping calculated by shifting the static component of the transverse resistance in different directions, which are indicated by solid arrows. The unit for *x* and *y* axis are in μm for all the panels.

Figure 2a shows the static component of the 2D transverse resistance mapping of a platinum Hall bar, detected at the modulation frequency of the applied ac current, as the laser spot is scanned across the Hall bar. The illumination induces a localized hotspot, where the resistance increases due to heating. Consequently, the transverse resistance takes a positive or negative value depending on the position of the localized hotspot. The transverse resistance is positive if the laser is scanning inside the R1 and R4 regions and negative in the R2 and R3 regions (Fig. 2a).

The 2D mapping of the transverse resistance produced by heating with the laser spot (which can only increase the resistance) allows to foresee the symmetry of the transverse photoresistance generated by the helicity variation (which can be both positive and negative). Because of the Wheatstone bridge geometry, in the Hall bar the spin accumulation, depicted in Fig. 1a, produces a contrast with a different symmetry. In Fig. 2f, we simulate the resistance mapping expected from the pure HPR effect, which is expected to be proportional to the curl of the electric current (see Supplementary Information Note 5.2).

Similarly to the measurements on the ring-shaped device, we can now compare the HPR signals to the beam shift effect. We estimate the beam shift-induced photoresistance by subtracting the static resistance mapping from a slightly shifted version of itself. This procedure mimics exactly the experimental beam shift effect. As shown in Fig. 2g–j, the resistance mapping depends strongly on the beam shift direction. The calculations show that the beam shift produces a resistance change with a different symmetry than the HPR mapping. To vary experimentally the beam shift, we rotated the sample while keeping the optical settings unchanged. Contrary to expectations for HPR, we observe that the photoresistance depends strongly on the orientation of the sample as shown in Fig. 2b–e. These results are well reproduced by the calculated resistance mappings corresponding to a constant beam shift. Within the precision of our measurements, no other measurable signal is observed in these experiments, in agreement with the result obtained in the ring-shaped devices. Our conclusion is that the beam shift signal also dominates in the transverse configuration.

The 2D transverse resistance mapping turned out to be a sensitive method for detecting the existence of beam shift-induced resistance modification and for determining the dominant component. To confirm our conclusions, we measured 2D mappings for thin films of Au, Ta, Cu, Pt/Co/Pt, Pt/Co/AlO_*x*_ on SiO_2_ substrate, as well as for a topological insulator layer Bi_2_Se_3_ on Al_2_O_3_ (sapphire) substrate. The results are shown in Supplementary information Note 5.3. We observe that the beam shift-induced resistance dominates in all our experiments. Moreover, since the beam shift signal has a trivial origin, we can precisely calculate its magnitude, and assess the possible presence of non-trivial components.

The transverse photoresistance is measured with high accuracy due to the Wheatstone bridge configuration. It should be noted that the method is sensitive not only to the beam shift but also to the power variation of the laser beam. This could create another component of the transverse signals that might have been neglected in the photoresistance or photocurrent experiments with the transverse configuration, e.g. in spin to charge conversion ratio estimation^19,20^. Since these effects have very different symmetries, we systematically detangle them by artificially distorting the modulated laser beam, as discussed in detail in Supplementary Information Note 3.

### Beam shift magnitude estimation

**Fig. 3:**
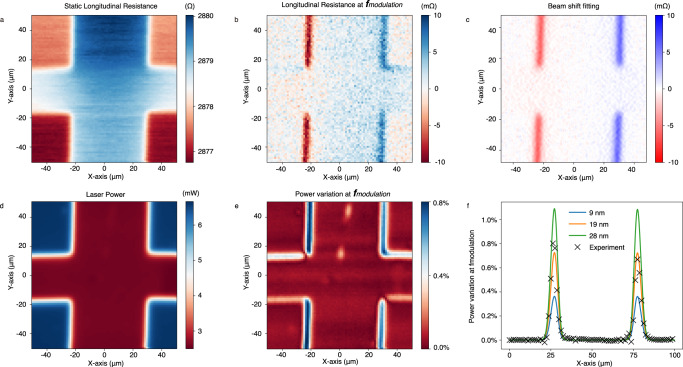
Beam shift estimation with beam shift induced resistance and power variation. **a** Static component of the longitudinal resistance mapping indicating the profile of the cross. When illuminating the device, the longitudinal resistance increases. **b** Longitudinal photoresistance measured at the laser modulation frequency *f*_modulation_. **c** Fitting result obtained by subtracting the line-scan resistance from a shifted (by 19 nm) version of itself. **d** Mapping of the static component of the transmitted laser power and **e** the power variation ratio measured at the modulation frequency. **f** Line scan of the laser power variation (dashed crosses) and the simulated power variation with different shift distances (9, 19, 28 nm). The best fit is obtained for the same shifting distance of 19 nm in perfect agreement with the result of the longitudinal resistance fitting.

Now that we have established the prevalence of the beam shift related signal over the HPR, we can estimate the magnitude of the beam shift. From the static longitudinal resistance mapping, we calculate the beam-shift effect (for different shift values) and compare it to the experimental result (Fig. 3). We choose the direction with the largest beam shift signal. For this particular device orientation, we evaluated the beam-shift to be 19 nm. The details of the fitting can be found in Supplementary Information Note 4. We further verify this procedure by performing measurements of the absorption of the laser beam within the same experiment. In this case, we extract the beam shift by comparing the transmitted static optical power and the optical power at the fundamental modulation frequency. Once again, we find a beam shift of 19 nm, in perfect agreement with the resistivity measurements. Note that this beam shift is two orders of magnitude smaller than the laser spot diameter (1.7 µm). Therefore, it cannot be visualized in a wide-field imaging of the device, which is typically used for the optical setup alignment.

The beam shift-induced photoresistance is also non-trivial when then beam diameter is large (e.g., an unfocused Gaussian beam from the laser source), for which both the width and the position of the device affect the result, as can be seen from the Supplementary Note 7.

It should be pointed out that the laser beam shift is not exclusive to a single experimental setup. We used different laser diodes with wavelengths of 532, 790, 960, and 1040 nm, as well as different polarization modulators. The beam shift phenomenon always exists, varying between 5 and 25 nm depending on the setup. The beam shift photoresistance exhibits a linear relationship with the illumination power, similarly to the HPR (Supplementary Information Note 8).

To understand why this effect can be so prevalent, we made estimates of the beam shift values based on the specifications of commercial optical components. We implicitly assumed that the optical setup is perfectly aligned and the only beam shift contribution comes from the imperfections of the birefringent crystal (Supplementary Information Note 1). These estimated beam shifts are of the same order of magnitude as our experimental observations (up to tens of nm). This indicates that in most cases the effect of beam shift cannot be removed simply by adjusting the alignment of the different components. It can be reduced by using high quality optical components in combination with short working-distance objectives. However, long working-distance is often advantageous for experiments involving electric measurements to accommodate for electrical connections, while in low-temperature experiments, the long working-distance is typically imperative.

**Fig. 4:**
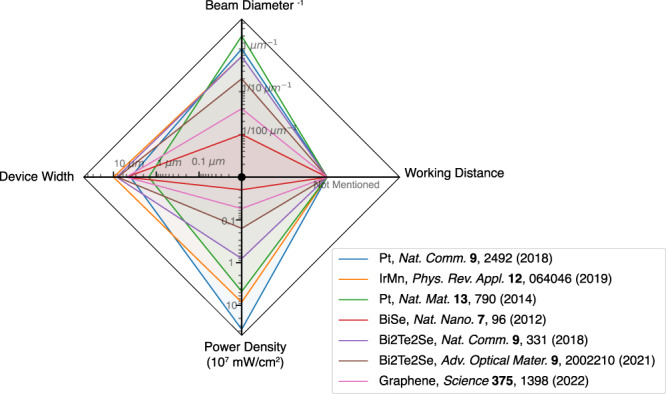
Graphical representation of the experimental parameters driving the beam shift-induced photoresistance. Different material systems including metals, topological insulator, semimetals are selected from published works. The laser spot diameter, device width, and laser power are radar plotted for comparison. It is important to note that the working distance used in these experiments is not mentioned. For this reason, we assigned the same value for all. The beam-shift effect is expected to be larger as the four parameters plotted in this graph increase.

In Fig. 4 we included a graphical representation of some of the experimental parameters influencing the beam shift, as reported in several works. Different material systems such as conventional metals, topological insulators, 2D Dirac semimetals are selected. The beam diameter, device width, laser power, and wavelength, are all very different in these experiments. The beam shift-induced photoresistance is related to (a) The longitudinal resistance enhancement during illumination, which is also highly material dependent and wavelength dependent; and (b) The beam shift distance. For the beam shift distance during the measurement, the two most important factors are: (1) the effective parallelism of the helicity modulator; and (2) the working distance of the focusing objective, or in the case of un-focalized light, the distance from the helicity modulator to the device. Furthermore, for a given beam shift distance, a smaller ratio of the beam diameter/width of the device produces a larger beam shift-induced photoresistance. Among the references in Fig. 4, the effect of the beam shift-induced photoresistance is not mentioned in the manuscript, nor the working distance/parallelism of the illumination system. Consequently, this plot should not be regarded as an assessment of the quality of other works, but rather as a tool to recognize the most significant parameters affecting the beam-shift effect in different experiments.

To conclude, we observed beam-shift-related effects in the helicity-dependent photoresistance measurement, regardless of the particular device or experimental geometry. We find that the beam shift-induced effect dominates the measured signals, and we do not find any trace of helicity-dependent photoresistance. The beam shift effect discussed in our work should be estimated and corrected in all experiments where the circularly polarized laser beam is used to locally influence the electrical properties (i.e., helicity-dependent photocurrent or photoresistance). The effect of the beam shift is not properly documented in any of the existing studies that we are aware of. The previous experiments should be revisited and possibly corrected for this effect before making any conclusions about the coupling between the circularly polarized light and spin polarization or transport, and before extracting any numerical value for any related physical parameter.

## Methods

### Measurement

We employed laser beams of several fixed wavelengths between 532 and 1040 nm, which were normally incident on the device. The focused spot size ranged from 1 to 4 μm, and we varied the laser power from 1 mW to 80 mW. The data in the article is with a 15 mW 532 nm laser. Three types of polarization modulators were used to modulate the helicity of circular polarization of the light at frequencies from 77 Hz to 50 kHz (*f*_light_): 1. Photoelastic Modulator from Hinds Instruments Model PEM-100; 2. Electro-Optic Modulator from Thorlabs Model EOAM-C4; 3. Linear polarization liquid crystal retarder from Meadowlark Optics Model LVR100, in combination with a λ/4 plate from Edmund Optics (12.7 mm Dia. 532 nm λ/4 Quartz). Each polarization modulator was first calibrated. We inserted a polarizer between the detector and the modulator, and then monitored the real-time waveform of the output power. The transmitting axis of the polarizer is set to be parallel to the intermediate (linear) polarization direction, generated between the left hand and right hand circular polarized light. After fine-tuning the input signal of the modulator, the output waveform exhibited a sinusoidal waveform with a frequency of 2×*f*_light_. Then the modulator was mechanically adjusted on a six-axis precise positioning stage. We first used a conventional far-field reflection beam correction method, by overlapping the reflected beam position to its reflection point for each optical component, with a total optical path distance of 1.5 meters. The position of the modulator was further corrected by minimizing the magnitude of the FFT spectra for the transmitted laser power at the fundamental modulation frequency for a highly transparent sample. For less transparent samples, instead of the transmitted laser power, we monitored the transverse photoresistance at the fundamental modulation frequency. Details of this procedure are discussed in Supplementary Information Note 2. An AC signal at the frequency of 10 Hz (*f*_current_) was applied to the device, with the amplitude of 5 V for the Hall bar devices and 10 V for the ring shape devices. The applied AC current and the laser modulation signal were synchronized by a NI-PXI system or two SRS 830 lock-in amplifiers, acting as a dual frequency lock-in detection. A resistor was connected in series to the device to detect the drift current amplitude and the longitudinal resistance change induced by the circularly polarized light. The photoresistance signal was measured at the frequency of *f*_light_ + *f*_current_. The longitudinal and transverse resistances measured at *f*_current_ reflect the static electric properties, referred to as static resistance in this article. A fast silicon optical detector was placed behind the sample (without any optical components in between) to monitor the real-time power variation of the transmitted laser beam. The sample was set on an *x*-*y* axis stepper motor stage for scanning. All the measurements were performed at room temperature.

### Sample fabrication

The metals were deposited, either by DC sputtering or E-beam evaporation, on a transparent glass substrate. The deposition was followed by a two-step photolithography process to fabricate the devices and remove the photoresist at the end of the process. The thickness of Pt is 3 nm for ring-shaped devices and 6 nm for Hall bar devices. Both sputtering and E-beam evaporation were used for Pt film deposition, and no clear difference in photoresistance is found for the different deposition methods. Au (5 nm, evaporation), Pd (5 nm, evaporation), Ta (5 nm, sputtering), Cu (5 nm, sputtering), Pt/Co/Pt (3 nm/0.6 nm/1.8 nm, sputtering), Pt/Co/AlO_x_ (3 nm/3 nm/2 nm, sputtering) were deposited on SiO_2_ substrate. 10 nm thick Bi_2_Se_3_ is grown on sapphire substrate by molecular beam epitaxy in the van der Waals regime^39^. The details of the fabrication can be found in our previous publications^40^. The size for the Hall cross device is 50 μm × 30 μm for metallic films, and 10 μm × 10 μm for the topological insulator. The ring-shaped device has a width of 30 μm and a diameter of 200 μm.

## Supplementary information

Supplementary Information

## Data Availability

The authors declare that all the data used to reach the conclusions, and necessary to reproduce the results, is presented in the manuscript and the supplementary information.
