# Supplementary material for: Helicity dependent photoresistance measurement vs. beam-shift thermal gradient
Source: Nat Commun. 2022 Nov 10;13:6790. doi: 10.1038/s41467-022-34198-5 (PMC9649656; doi:10.1038/s41467-022-34198-5)
Supplement: Supplementary file 1 — Supplementary Information [file 41467_2022_34198_MOESM1_ESM.pdf]

Supplementary information

Helicity dependent photoresistance measurement vs. beam-shift thermal gradient

Haozhe Yang[1*†], Eva Schmoranzerova[1,2], Pyunghwa Jang[1], Jayshankar Nath[1], Thomas Guillet[1], Isabelle Joumard[1], Stéphane Auffret[1], Matthieu Jamet[1], Petr Němec[2], Gilles Gaudin[1], Ioan Mihai Miron[1*]

1. Univ. Grenoble Alpes, CNRS, CEA, SPINTEC, F-38000 Grenoble, France.

2. Faculty of Mathematics and Physics, Charles University, Czech Republic.

† Present address: CIC nanoGUNE BRTA, 20018 Donostia-San Sebastian, Basque Country, Spain

* Correspondence to: haozheyang.cnrs@gmail.com, mihai.miron@cea.fr

Note 1 Beam shift tolerance calculated with commercial optics components.

Note 2 Laser beam and modulator adjustment based on the transverse photoresistance analysis.

Note 3 Quantitative analysis of the mixture effect between beam shift induced photoresistance and spin-related helicity dependent photoresistance.

Note 4 Beam shift distance estimation with longitudinal resistance.

Note 5 Results for different films and substrates.

5.1 Longitudinal photoresistance results for ring shape device for different metals/substrates.

5.2 Simulation of the transverse HPR signal.

5.3. Transverse photoresistance results for different metallic samples and substrates.

5.4. Transverse photoresistance results for topological insulator Bi_2Se_3.

Note 6 Measurement illustration and helicity dependent longitudinal resistance estimation using SMR model.

Note 7 Beam shift induced photoresistance calculation with large beam size.

Note 8 Photoresistance as a function of laser power with different wavelengths for Pt and Cu.

References.

Note 1 Beam shift tolerance calculated with commercial optics components

The helicity-dependent photoresistance or photocurrent experiment can use either a quarter-wave plate (λ/4) or photoelastic modulator. We already estimated the beam shift distance for the photoelastic modulator to be 19 nm in a particular case. In this note we will make estimates of the unintentional beam shift that can result from the quarter waveplate rotation, in a laser scanning experiment with commonly used commercial optical components.

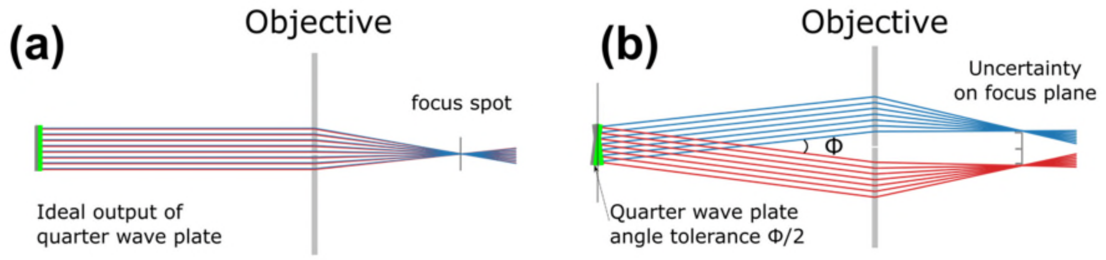

Supplementary Figure 1 (a) In the case of perfect alignment of a perfect quarter-wave plate, the beams obtained for different angular orientations of the wave plate are focused in the exact same position. (b) Due to optical refraction, when the wave plate is not perfectly coplanar, the two collimated beams corresponding to two orientations of the wave plate deviate in different directions. The beam deviation is linked to the wave plate planarity. The tolerance value for this parameter is generally provided by the supplier of the optical components.

The illustration for an ideal case is shown in Supplementary Figure 1 (a). The two beams (blue and red) are focused on one spot. If we insert an imperfect quarter waveplate that creates a beam deviation (Φ), these two collimated beams will reach the objective with a different angle, and focus to a different position Supplementary Figure 1 (b). We estimated the beam shift uncertainty with three commercial quarter waveplates from THORLABS (WPQ10E Polymer Waveplate), MKS New Port (05RP04 Quartz Waveplate), and Edmund Optics (λ/4 Quartz Waveplate Zero Order), and two objectives from Nikon (CFI60 TU Plan Epi ELWD11 50X) and Olympus (SLMPLN WD18 50X). Considering the scanning experiment in normal environment (air), when the beam deviation is small, the beam shift distance d is estimated with $d = \sin\Phi \times f0 \approxeq \Phi \times f0$ where f0 is working distance of the objective. The results are shown in Supplementary Table 1. We can find that the smallest beam shift distance is 27 nm of the same order of magnitude as in our experiments. These results indicate that the effect of beam shift induced photoresistance or thermal current should also be considered when using the rotating quarter wave plate configuration.

We note that the beam shift can be reduced by using objectives with shorter working distance. However, this is impractical for experiments where an electrically connected samples is placed on a scanning stage. In such experiments, it is most common to use a relatively larger working distance objective, which leaves enough free space for the electrical connections.

Supplementary Table 1 Beam shift distance estimated with three commercial quarter waveplates and two objectives

	**Thorlabs® WPQ10E Polymer Waveplate** Parallelism < 5 arcsec	**MKS Newport® 05RP04 Quartz Waveplate** Parallelism <1 arcsec	**Edmund Optics® λ/4 Quartz Waveplate Zero Order** Parallelism <0.5 arcsec
Nikon® CFI60 TU Plan Epi ELWD11 50X	266 nm	53 nm	27 nm
Olympus® SLMPLN WD18 50X	432 nm	84 nm	42 nm

Note 2 Laser beam and modulator adjustment based on the transverse photoresistance analysis.

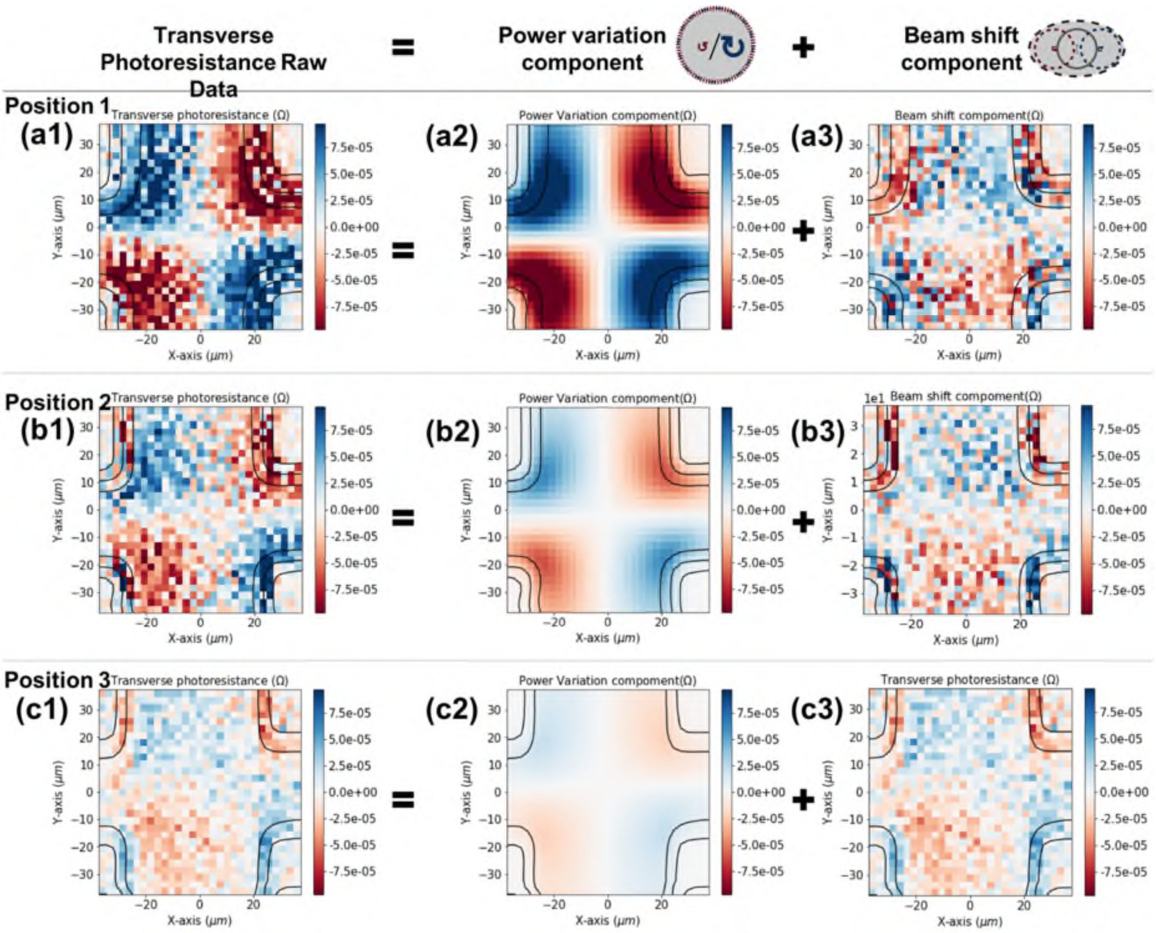

Supplementary Figure 2. Transverse photoresistance 2D mapping for Pt Hall cross with three positions of the photoelastic modulator. The raw data for position 1 (a1), position 2 (b1), position 3 (c1). Using symmetry analysis, the raw data could be split into two component. The first one, created by the power variation associated the helicity reversal, is shown in the second column (a2), (b2), and (c2). The other one is the beam shift induced photoresistance, shown in the third column (a3), (b3) and (c3).

The laser beam is adjusted carefully before every experiment. We first use the conventional far-field laser beam adjustment method, to confirm the beam have a vertical incidence angle towards each optical component.

In a second step, we fine-tune the lateral position of the polarization controlling component (photoelastic modulator in this example), which we found empirically to be one of the critical parameters in our setup.

Since the Hall bar is extremely sensitive to detecting the beam shift (it is equivalent to a Wheatstone bridge – see main text), we use it as a tool for adjusting the optical setup. As we adjust the setup we observe two types of variations: i) the difference in total laser power for the left and

right-handed circular polarizations. This power variation creates an AC resistance variation with a similar mapping as the DC signal from the laser spot induced heating. ii) the second contribution is linked to the beam shift-induced photoresistance, as discussed in the main text.
In Supplementary Figure 2, we show three examples of results obtained (named Position 1, 2, and 3 of the photoelastic modulator) during the alignment process. One could find that the amplitude of the transverse photoresistance decreases from Position 1 to 3, as shown in Supplementary Figure 2 (a1) (b1) and (c1). We can separate the power variation component and the beam shift component (Supplementary Figure 2) using the fact that they have a different symmetry. We subtract the renormalized DC resistance in order to remove entirely the AC resistance variation. The remaining signal is due to the beam-shift. Our empirical optimization of optical alignment is guided by the minimization of these two components.

Note 3 Coexistence between beam-shift induced photoresistance and spin-related helicity dependent photoresistance.

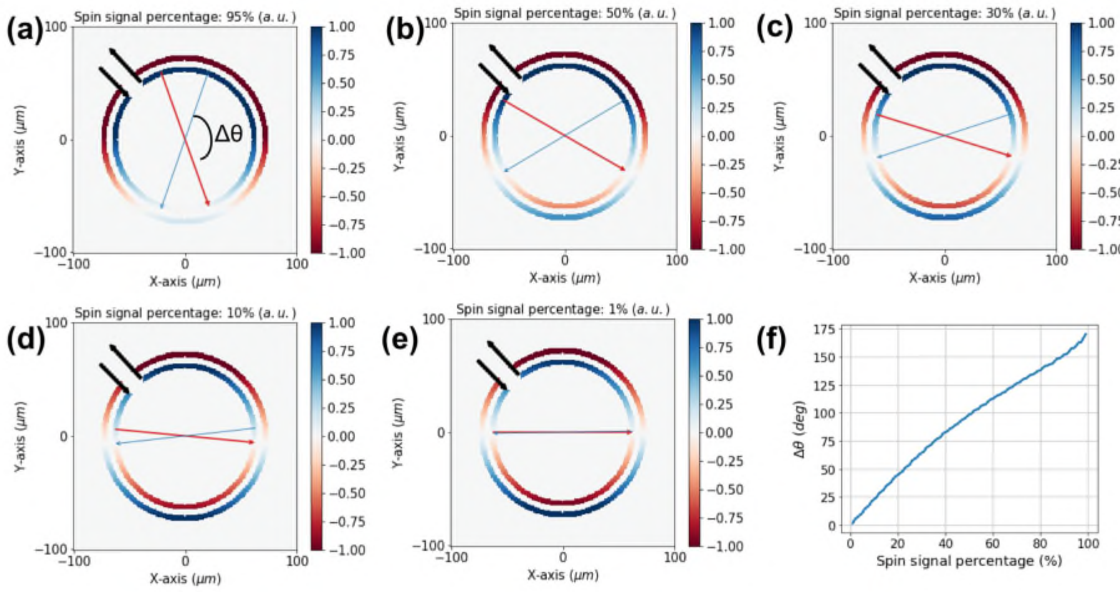

Supplementary Figure 3 Numerical simulation showing the mixing of the beam shift induced signals with the helicity dependent spin signal in different proportions (95% (a), 50% (b), 30%(c), 10%(d), and 1%(e)). The black arrow indicates the current direction. Red and blue arrows travel through the center of the ring and point to the signal crossing the zero point. Δθ indicates the angle between the zero photoresistance position and the diameter across the other side of the zero photoresistance position (red and blue arrow). (f) Numeric calculation result for spin signal percentage vs. Δθ.

When the photoresistance is a mixture of helicity dependent spin signal and beam shift induced signal, the 2D resistance mapping of the photoresistance for the ring shape device will be in some intermediate state between Fig.1 (b) and (e). We simulate the variation of the percentage of spin signal in the total resistance mapping. We plotted 4 different 2D mappings with 95%, 50%, 10% and 1% percentage of spin signal in Supplementary Figure 3 (a)-(d). The blue and red arrows cross the center of the ring and point to the zero photoresistance points. The angle Δθ between the two arrows measures the magnitude of the spin signal. The simulation result is shown in Supplementary Figure 3 (f), showing how the angle Δθ increases when increasing the percentage of the spin signal. Another signature of the spin signal is the asymmetry of the maximum intensity. When the spin signal increases and the zero-crossings approach each-other, the signal maximum and minimum values on one side of the circle are different than on the other side. These are the two indicators of the spin signal that we will observe in the experimental data.

Note 4 Beam shift estimation based on the variation of the longitudinal resistance.

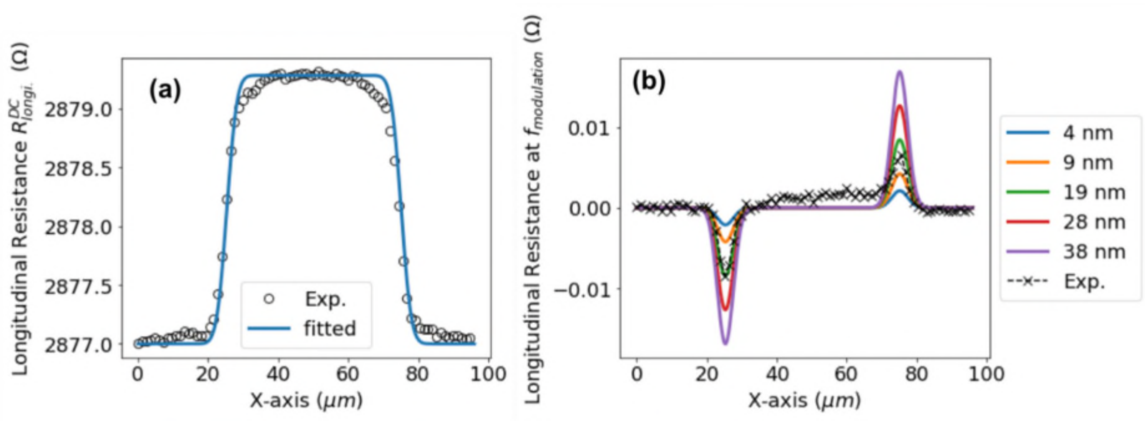

Supplementary Figure 4 (a) Measurement of the longitudinal resistance during a line scan of the laser spot across the device (circles). The calculated DC resistance is plotted in solid lines. (b)Measured longitudinal resistance at the modulation frequency, with different beam shift calculated results (solid lines).

The measured longitudinal resistance of the device is fitted with two Gauss functions $R_{DC}^{ini} = \frac{A}{\sigma\sqrt{2\pi}}\int_{-\infty}^{+\infty}\left(e^{-\frac{1}{2}\left(\frac{x-x_1}{\sigma}\right)^2} + e^{-\frac{1}{2}\left(\frac{x-x_2}{\sigma}\right)^2}\right)dx + R0$. where x is the position of the laser spot, x_1 and x_2 correspond to the position of the left edge and right edge of the device, A is magnitude, σ is related to the FWHM according to $FWHM = 2\sqrt{2\ln 2}\sigma$, R0 is the longitudinal resistance without illumination.

As we introduce the laser spot shifting of Δx from the initial position, the equation of the longitudinal resistance R_{DC}^{ini} then converts to $R_{DC}^{final} = \frac{A}{\sigma\sqrt{2\pi}}\int_{-\infty}^{+\infty}\left(e^{-\frac{1}{2}\left(\frac{x-x_1+\Delta x}{\sigma}\right)^2} + e^{-\frac{1}{2}\left(\frac{x-x_2+\Delta x}{\sigma}\right)^2}\right)dx + R0$. The resistance difference $\Delta R = R_{DC}^{ini} - R_{DC}^{final}$ reproduces the beam shift induced longitudinal resistance. It matches the resistance measured experimentally at the polarization modulation frequency, as shown in Supplementary Figure 4 (b), where different ΔR curves with Δx of 4 to 38 nm are illustrated in solid lines. The laser beam shifting distance Δx is fitted to be 19 nm.

Note 5 Results for different films and substrates

5.1 Longitudinal photoresistance results for ring shape device for different metals/substrates.

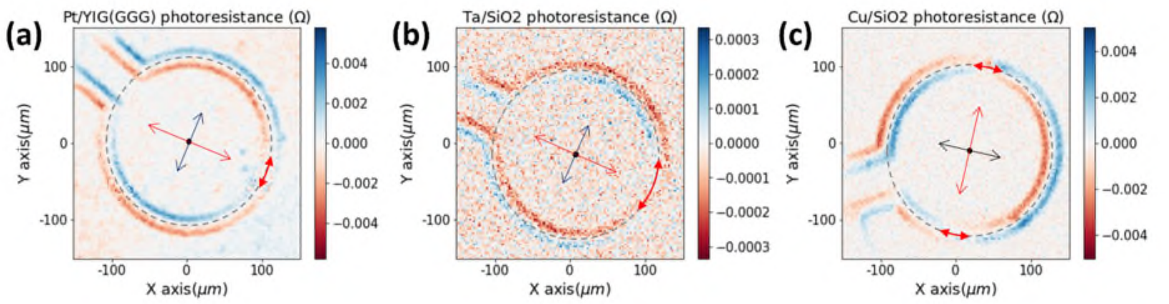

Supplementary Figure 5.1 Longitudinal resistance 2D mapping for (a) Pt on YIG/GGG substrate, (b) Ta on glass substrate, and (c) Cu on glass substrate. All the results obtained on all the different samples exhibit the characteristic features of the beam shift induced signal. The red arrows point towards the position of the zero crossings, the red arc circle indicates the approximate uncertainty of the position of the zero crossing. The black arrows point towards the position of the maximum intensity.

Depending on the direction of the beam-shift, the zero-crossing position can fall onto the position where the current is entering structure, and where the circular path is distorted. In this case, the zero-crossing positions cannot be used to assess the presence of the HPR signal, but we can rely on the maximum intensity values. As explained in Note 3, the two signal intensity maxima should differ in the presence of HPR. We do not observe any measurable asymmetry that could support the presence of HPR in any of our measurements, in any of our samples.

5.2. Simulation of the transverse HPR signal.

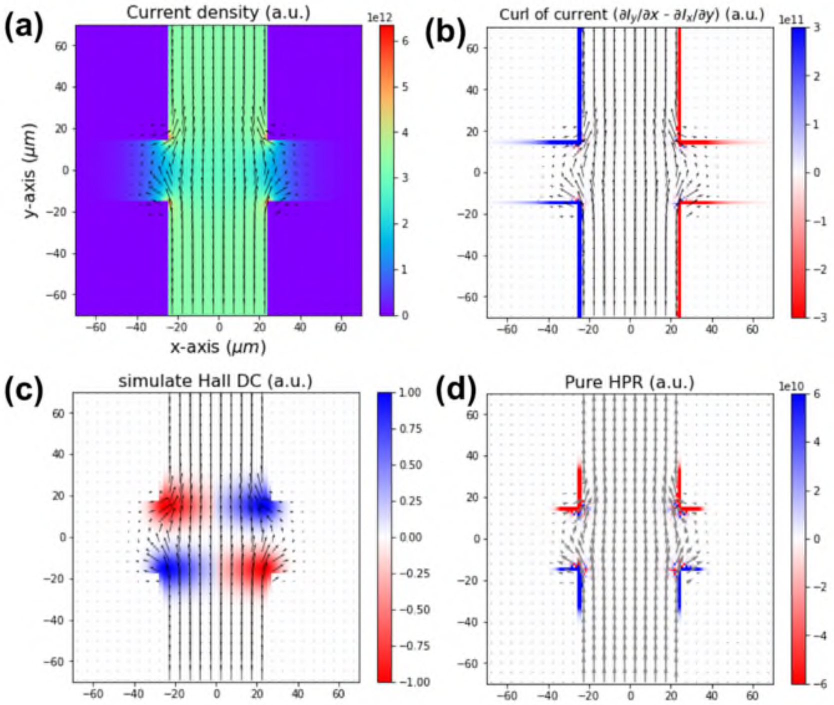

Supplementary Figure 5.2a) Numerical simulations of electrical detection of the HPR in the Hall cross device. (a) direction (arrows) and intensity (color scale) of the current intensity. (b) calculation of the curl of the electric current. (c) sensitivity map of the Hall bar to local changes of the resistance. (d) calculation of the HPR signal as a convolution between the gradient of the electric current and the device sensitivity.

The creation of the spin accumulation that underlies the HPR effect, is expected to be proportional to the curl of the electric current. This is why, the spin accumulation is largest on the edges of the current path. The measured transverse HPR effect is a convolution of the spin accumulation and the electrical sensitivity Hall cross. For this reason, the resulting signal has a different symmetry than in the simpler geometry of the longitudinal detection. It is also important to notice that the symmetry of the HPR is different than the symmetry of the beam-shift signal (described in the main text).

5.3. Transverse photoresistance results for different metallic samples and substrates.

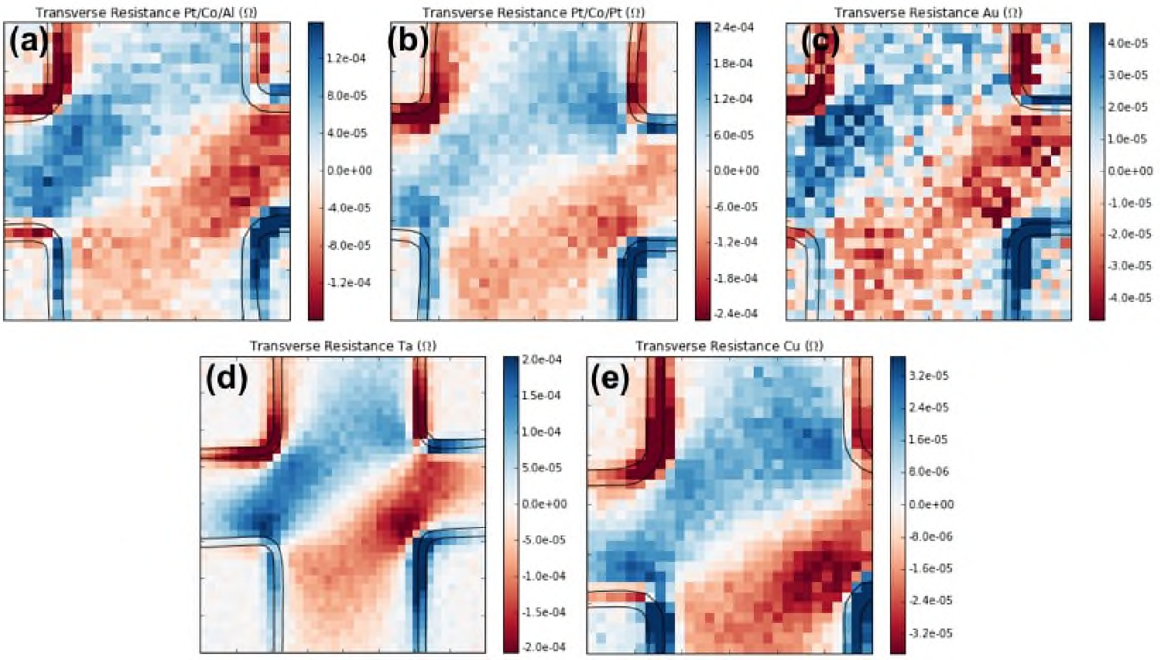

Supplementary Figure 5.3 Transverse resistance 2D mapping for (a) Pt/Co/AlO_x (3 nm/3 nm/2 nm, sputtering), (b) Pt/Co/Pt (3 nm/0.6 nm/1.8 nm, sputtering), (c) Au (5nm, Ebeam evaporation), (d) Ta (5 nm sputtering), (e) Cu (5 nm sputtering). All the samples exhibit the beam shift features in the 2D mapping.

The characteristics of the HPR effect, only occurring on the edges of the sample, and its specific symmetry (supplementary note 5.2) and are not observed in any of the different samples that we have tested. It appears that the HPR is much weaker than the beam-shift signal.

5.4. Transverse photoresistance results for topological insulator Bi_2Se_3.

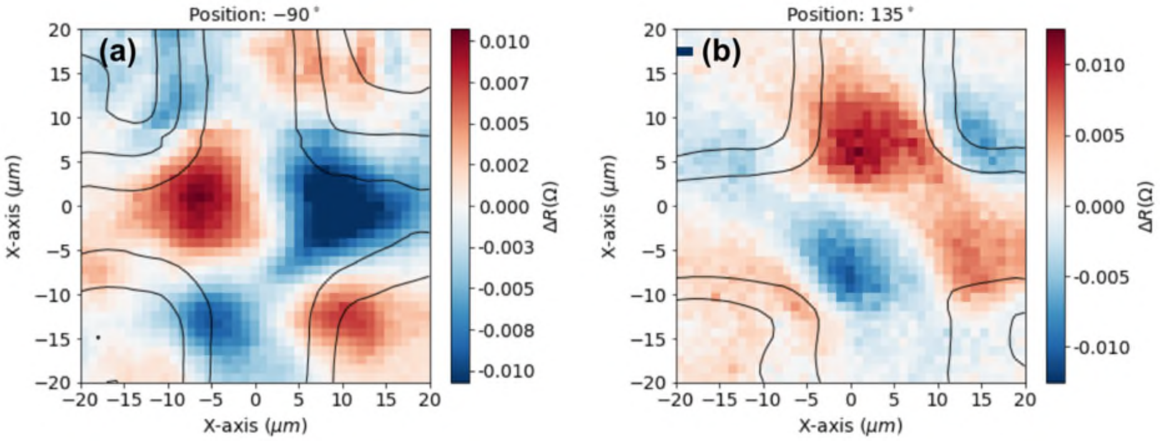

Supplementary Figure 5.4 Transverse resistance for Bi_2Se_3/Sapphire measured at the laser modulation frequency with changing the in-plane orientation of the cross with respect to the optical setup: (a)-90 deg and (b) 135 deg. The width of the Hall cross is 5 μm. Once again, for both orientations we observe the 2D mapping pattern produced by the beam shift induced photo resistance.

The topological insulator thin film was patterned into a smaller sized Hall bar, compared to the metallic samples. Once again, the experimental signal is very different than the signal expected from the HPR. Moreover, because the shape of the Hall cross is not as well defined as for the metallic films, we also verify the origin of the signal by rotating the sample. After rotation, the signal changes entirely, proving that the HPR contribution is much smaller than the beam-shift signal.

Note 6 Measurement illustration and helicity dependent longitudinal resistance estimation using SMR model.

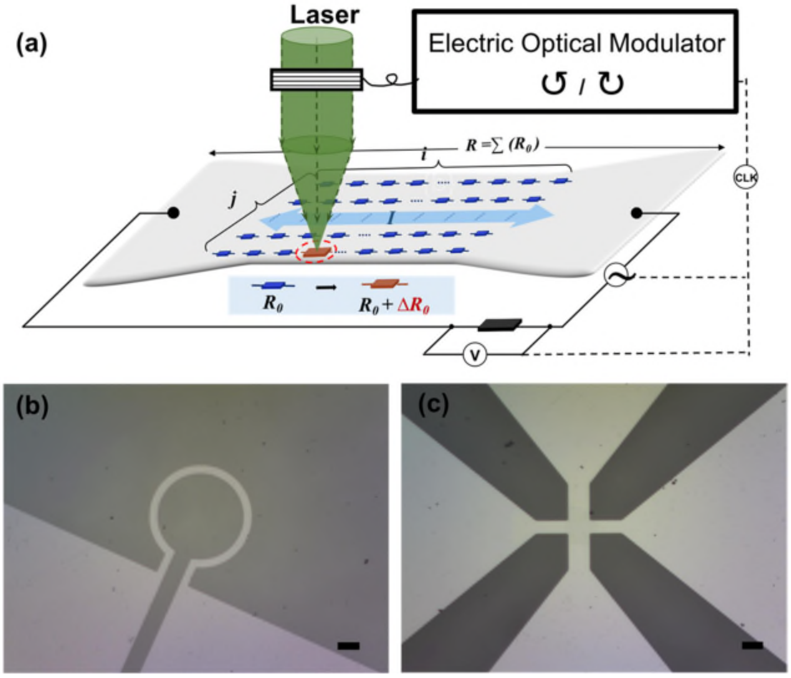

Supplementary Figure 6.1 (a) Sketch of helicity dependent photoresistance measurement using a scanning laser with alternating helicity. The longitudinal resistance is measured at the frequency of the polarization modulator. The sample can be considered as formed of an array of $i \times j$ resistor elements with initial resistance of R_0. As the illuminated elements change their resistance ($R_0 + \Delta R_0$) the total longitudinal resistance becomes $R + \Delta R$. Optical image of the device used in the main paper for (b) ring-shape device and (c) Hall cross device. The scale bar represents 50 µm.

The measurement setup (a) and optical image for ring shape device (b) and Hall bar device (c) are illustrated in Supplementary Figure 6.1, along with a matrix resistor model for the helicity dependent longitudinal resistance. The length of the bar is l, and the width is w. The bar, consisting of $i \times j$ matrix of resistors, has the total resistance of R. Each element resistor has the initial resistance R_0. When the circularly polarized light illuminates the edge of the sample, the elementary resistor changes to $R_0 + \Delta R_0$, where ΔR_0 is the resistance change due to the spin magnetoresistance. We define $\Delta R_0 = k_{SMR} \cdot R_0$, where k_{SMR} indicates the spin magnetoresistance ratio induced by circular light. In this toy model, we consider that when the laser illuminating the bar, only one resistor changes its value while others stay constant. So the longitudinal resistance change of the bar would be

$$\Delta R = \frac{1}{\frac{1}{R_0 + \Delta R_0} + \frac{j-1}{R_0}} - \frac{R_0}{i}$$

The length of each resistor along the x-direction should be the diameter of the laser spot d. The spin accumulation can only exist within a distance smaller than the spin diffusion length from the edge of the device. Therefore, we can define the width of the elementary resistor to be the spin diffusion length λ. Then we obtain $i = \frac{l}{d}$, $j = \frac{w}{\lambda}$. Considering the resistivity ρ of the material and thickness t, the total resistance can also be expressed as $\frac{l}{wt}$. Since the elementary resistor becomes $R_0 = \frac{\rho d}{t\lambda}$, the overall resistance of the bar change due to the circular light illumination is

$$\Delta R = \frac{\rho d \lambda}{t w^2 (1 + 1/k_{SMR} - \lambda/w)}$$

The measured helicity dependent resistance change of the bar is 2×10^{-3} Ω. If we use the $k_{SMR} = 2 \times 10^{-4}$[1], which is the spin magnetoresistance of Pt and CoFeO. The resistivity ρ = 310 μΩ cm, and the spin diffusion length λ to be 5 nm [2], the resistance change for this device should be $3.7 \times 10^{-7}\Omega$, which is much less than the measured value $2.0 \times 10^{-3}\Omega$.

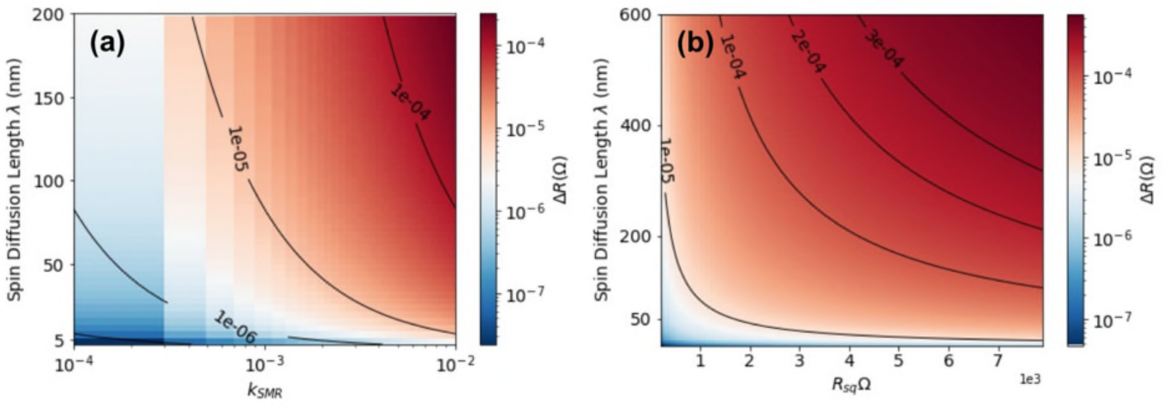

Supplementary Figure 6.2 Calculated helicity dependent photoresistance ΔR as function of (a) spin diffusion length λ and SMR ratio k_{SMR}; (b) spin diffusion length λ and square resistance R_{sq}.

However, the spin diffusion length and SMR ratio could differ[3,4], which then could have an influence of the helicity dependent photoresistance value. We plot the calculation result by changing the spin diffusion length from 5 nm to 200 nm, and the SMR ratio from 1×10^{-4} to 1×10^{-2}. The results are shown in Supplementary Figure 6.2 (a). Three solid contour lines correspond to helicity dependent photoresistance of10^{-8} Ω, 10^{-7} Ω, 10^{-6} Ω. Also, we plot the helicity dependent photoresistance as a function of spin diffusion length and the square resistance $R_{sq} = \frac{\rho}{t}$, as shown in Supplementary Figure 6.2 (b). We find that the calculated value is still far less than the measured data even if we choose a huge spin diffusion length and SMR ratio.

Note 7 Beam shift induced photoresistance calculation with large beam size.

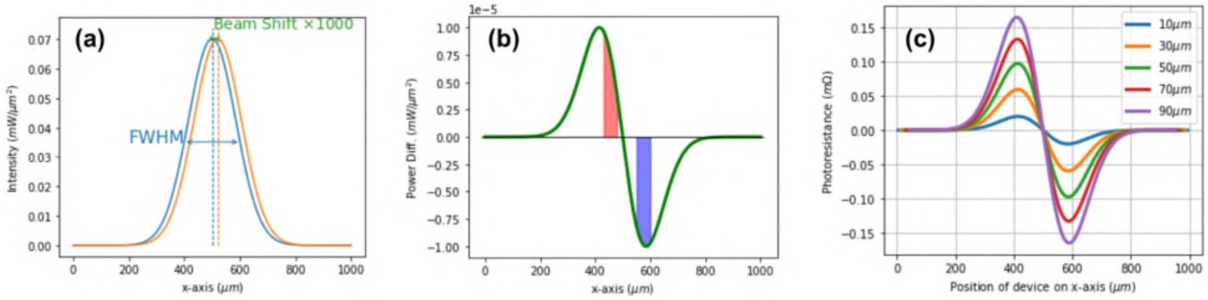

Supplementary Figure 7 (a) Calculated 1D Gaussian beam profile with a fixed width of 200 µm. For the following calculations we will use a 20 nm beam shift, which we make visible in (a) by magnifying it 1000 times. When a device (50µm wide) is placed under a large beam that shifts by a small amount, it experiences an oscillatory power density as plotted in (b). If the sample is placed on the left side of the beam (depicted in red) or on the right side (blue) the beam shift signal does not have the same sign. In (c) we plot the beam shift induced photoresistance as a function of device position for different widths (from 10 µm to 90 µm). For all these calculations, we used the same value of the laser power (15 mW); the illumination-induced longitudinal resistance enhancement was set arbitrarily tor 3 Ω/ 15 mW.

Note 8 Photoresistance as a function of laser power with different wavelengths for Pt and Cu.

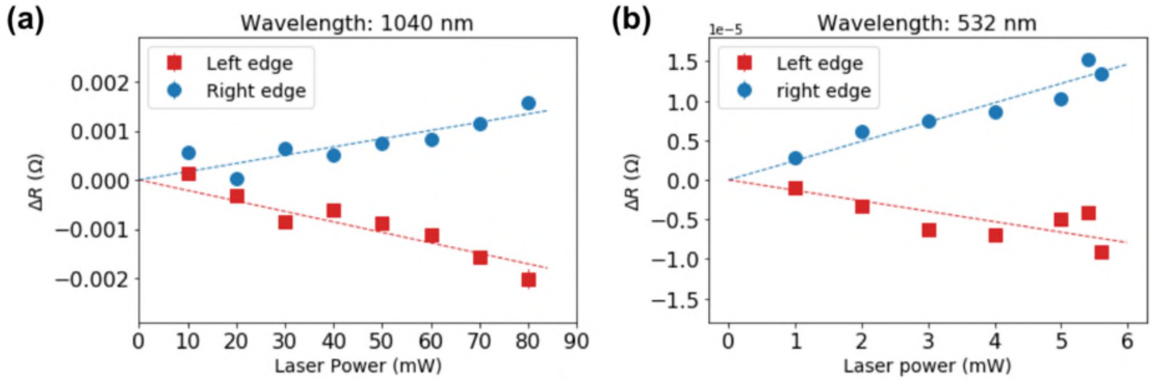

Supplementary Figure 8 Photoresistance ΔR for Hall bar devices as a function of input laser power for (a) Pt with 1040 nm laser, (b) Cu with 532 nm laser. The dotted lines are linear fitting results for the resistance increase at the two edges, indicating the beam-shift induced photoresistance exhibits a clear linear relation with the illumination power. The experiments for (a) and (b) were measured with two different setups.

Supplementary References:

1. Isasa, M. *et al.* Spin Hall magnetoresistance at Pt/CoFe2O4 interfaces and texture effects. *Appl. Phys. Lett.* **105**, 142402 (2014).
2. Ghosh, A., Auffret, S., Ebels, U. & Bailey, W. E. Penetration depth of transverse spin current in ultrathin ferromagnets. *Phys. Rev. Lett.* **109**, 127202 (2012).
3. Nakayama, H. *et al.* Spin Hall Magnetoresistance Induced by a Nonequilibrium Proximity Effect. *Phys. Rev. Lett.* **110**, 1–5 (2013).
4. Hahn, C. *et al.* Comparative measurements of inverse spin Hall effects and magnetoresistance in YIG/Pt and YIG/Ta. *Phys. Rev. B - Condens. Matter Mater. Phys.* **87**, 1–8 (2013).
